# mIoT Slice for 5G Systems: Design and Performance Evaluation

**DOI:** 10.3390/s18020635

**Published:** 2018-02-21

**Authors:** Riccardo Trivisonno, Massimo Condoluci, Xueli An, Toktam Mahmoodi

**Affiliations:** 1 Huawei Technologies, 80992 Munich, Germany; riccardo.trivisonno@huawei.com (R.T.); xueli.an@huawei.com (X.A.); 2 Department of Informatics, King’s College London, London WC2R 2LS, UK; massimo.condoluci@kcl.ac.uk

**Keywords:** 5G networks, mIoT, network slicing, control and user plane optimization

## Abstract

Network slicing is a key feature of the upcoming 5G networks allowing the design and deployment of customized communication systems to integrate services provided by vertical industries. In this context, massive Internet of Things (mIoT) is regarded as a compelling use case, both for its relevance from business perspective, and for the technical challenges it poses to network design. With their envisaged massive deployment of devices requiring sporadic connectivity and small data transmission, yet Quality of Service (QoS) constrained, mIoT services will need an ad-hoc end-to-end (E2E) slice, i.e., both access and core network with enhanced Control and User planes (CP/UP). After revising the key requirements of mIoT and identifying major shortcomings of previous generation networks, this paper presents and evaluates an E2E mIoT network slicing solution, featuring a new connectivity model overcoming the load limitations of legacy systems. Unique in its kind, this paper addresses mIoT requirements from an end-to-end perspective highlighting and solving, unlike most prior related work, the connectivity challenges posed to the core network. Results demonstrate that the proposed solution, reducing CP signaling and optimizing UP resource utilization, is a suitable candidate for next generation network standards to efficiently handle massive device deployment.

## 1. Introduction

Upon completion of use cases and requirements analysis, telecommunication industry is now moving towards developing 5G solutions to deploy from 2020 onwards. Leveraging on architecture modularization, service-oriented interfaces and end-to-end (E2E) network slicing, 5G systems are expected to enable the integration of vertical industries, thus aiming at creating an ecosystem able to efficiently support the heterogeneous features of tree use case categories [1,2], namely enhanced Mobile Broadband (eMBB), massive Internet of Things (mIoT) and Critical Communications (CriC). Architecture modularization and network slicing [3,4] will allow the design, deployment and management of specific E2E communication systems, featuring ad-hoc Control Plane (CP) and User Plane (UP) solutions conceived and optimized around the performance and functional requirements of related use cases.

In this scenario, the attention devoted to mIoT has been constantly increasing over the past years [5]. Market reports and data traffic forecasts predicted the proliferation of a wide variety of applications and services, where the massive deployment of, e.g., sensors, meters, actuators, wearables and connected appliances, will lead to a 75% increase of mobile connectivity request by the end of the decade [6]. In parallel, research and standardization communities are focusing on the challenges of mIoT, specifically in terms of device energy consumption, scalability and ubiquity of access, communication security, mobility and Quality of Service (QoS). Notably, the great majority of contributions put emphasis on access network and physical layer [7,8,9,10]. Hence, a key research and standardization challenge, addressed by this paper, is to design an E2E architecture targeting mIoT specific requirements, overcoming the key limitations of E2E 4G systems, to become a suitable candidate solution for 5G mIoT network slice.

Quite unique compared to all related prior work (always focusing on Access Network improvements), to the best of authors’ knowledge, this paper proposes and evaluates a 5G E2E network slice solution for the mIoT use case. To address the challenge of massive access in the mIoT scenarios, numbers of research works have proposed novel scheduling algorithms [11]. Furthermore, scalability of solutions is one of the major challenges in solutions proposed for mIoT [12], which is well addressed in this paper. The closest to the contribution of this paper, a connection-less transmission concept, is in [13], where devices transmit packets without any bearer establishment. However, this solution also focuses solely on radio access network (RAN) rather than an E2E approach including the mobile core network.

Furthermore, the solution in this paper has been designed around the 5G reference architecture recently finalized by the 3rd Generation Partnership Project, i.e., 3GPP [14]. Specifically, in E2E perspective, we identify limitations relating to core network (CN) and hence we focus on novel solutions to handle massive connectivity at CN, on both CP and UP sides, ensuring an efficient resource utilization and satisfying the diverse QoS required by different device categories. The proposed solution challenges the 4G “Always-ON” connectivity model, and proposes an alternative for 5G mIoT network slice, still connection oriented. Concisely, our proposal is based on the introduction of Virtual Devices, aggregating the CP and UP in the CN of multitudes of physical devices (PhDs) with homogeneous QoS requirements and traffic patterns characteristics. Our solution is able to drastically reduce the CP signaling load and also improve the UP resource utilization as it requires fewer UP resources in the CN to handle mIoT traffic. In summary, our contributions can be summarized as follows:

- Novel proposal on mIoT network slice model that challenges the 4G “Always-ON” connectivity model;
- Thorough performance study of the proposed solution;
- Demonstration of scalability of proposed solution for increasing number of IoT devices in mIoT use cases;
- Reporting result on significant reduction in CP signaling and UP resource utilization.

The paper is structured as follows. Section 2 reviews key mIoT requirements and the envisaged deployment scenarios. Section 3 examines Long Term Evolution (LTE)/Evolved Packet Core (EPC) system, and highlights 4G intrinsic design flaws, suggesting way forwards in 5G perspective. Section 4 provides a detailed technical description of the proposed solution, tightly linked to the ongoing 3GPP standardization activity to ensure its applicability to real systems to be deployed beyond 2020. Section 5 presents a quantitative performance evaluation of our solution. Section 6 concludes the paper.

## 2. The Massive Internet of Things Use Case

### 2.1. mIoT Service Requirements

Requirements of massive machine-type communications (mMTC) and mIoT have been extensively analyzed (e.g., [1,6,15,16], just to mention a few). Some aspects, however, need to be revisited to justify the effort being made to design the E2E mIoT network slices (i.e., including access and CN) within 5G systems. The analysis provided (e.g., [16]) concludes with the identification of three main application scenarios: IoT (large number of devices with different complexity and non-time critical synchronous/asynchronous data), smart wearables (low complexity and high battery life devices, requiring high reliability and possibly high data rates) and sensor networks (high density of heterogeneous devices with massive connectivity requirements). Coherently, the research work presented in [6] highlights that mIoT services may impose any combination of bandwidth and delay requirements. Moreover, the variety of device types and deployment scenarios may lead to hard challenges in terms of energy consumption, ubiquity, scalability and mobility of the connectivity (The requirements on security, authentication, and authorization are defined in [16], and are out of scope of this work). Additionally, different radio connectivity models may include direct device-to-device communications as well as direct or indirect (e.g., via relay nodes) connections to access nodes. Such considerations suggest mobile broadband networks, possibly supporting different access technologies, are the best candidates (compared to, e.g., Wi-Fi, LoRa and other simplified technologies which should surely only target some service niches) to support mIoT. Additionally, heterogeneous and tight QoS requirements suggest a connection oriented solution should be favored compared to connectionless ones. These consolidated considerations, backed by extensive prior art, provide the rationale and the basic guidelines to the design and standardization of mIoT E2E architecture and procedures to be featured by 5G mIoT network slices: mIoT network slice should feature a mobile cellular access and an E2E connection oriented connectivity model supporting different QoS profiles, targeting different clusters or categories of heterogeneous devices.

### 2.2. mIoT Deployment Scenarios and Traffic Models

Together with the analysis of communication requirements of mIoT, it is also of critical importance to envision possible deployment scenarios (i.e., number and geographical distribution of user equipment and UEs) and reasonable traffic models for different categories of services. These estimations, albeit speculative, help identifying bottlenecks, design flaws and unsuitable resource dimensioning criteria of current communication systems.

Models commonly used by a wide proportion of related work, formalized in [17,18,19], consider a device density between 10^3^ and 10^5^ UE/km^2^, leading to up to 10^6^ devices per cell, obviously depending on the scenario (e.g., central, urban, and rural). As per device traffic patterns, models have been defined aiming at stressing the radio access network (RAN) capabilities. Synchronous and asynchronous device transmission is considered, predominantly device triggered, with different access distributions in time (e.g., uniform, and beta-distributed) over periods ranging from tens of seconds to tens of hours. The UP traffic generated is expected mostly on the uplink (UL), each device activation requiring the transmission of data ranging between 10^2^ and 10^4^ bits. A quantitative comparison between the figures provided in this subsection and those available from data traffic reports for deployed 4G networks highlights mIoT, even at its early stage, will require networks to handle number of connections 3–4 orders of magnitude higher than Mobile Broadband (MBB) use cases, while each connected device will lead to a data traffic 5–8 orders of magnitude lower than 4G smartphones [17]. The above quantitative estimation should be carefully considered in the design of an E2E architecture: it should be emphasized that a region covered by, e.g., 10^3^ cells (which would be very likely handled by a single CN instance), would lead to up to one billion registered mIoT devices, although the load per cell would be 1–4 orders of magnitude lower than those generated by 4G smartphones. This leads to an essential design consideration: if such expected deployment scenarios and traffic models will be proven to be accurate on field, legacy 4G architecture would be challenged not on Access Network, but rather on the Core Network side, where novel solutions will be required. 

## 3. Gap Analysis for 4G System

Service requirements, device deployment scenarios and traffic models, briefly illustrated in Section 2.1 and Section 2.2, can be easily combined to highlight the severe limitations by design of 4G systems.

### 3.1. The 4G EPS Bearer and Always-ON Connectivity

4G LTE/EPC packet communication system, properly denoted as Evolved Packet System (EPS), relies on a connectivity model based on the “EPS bearer” and the “Always-ON” concepts.

The EPS is designed to provide IP connectivity between a UE and a PLMN (Public Land Mobile Network) external Packet Data Network (PDN). This service supports the transport of traffic flow aggregate(s), consisting of one or more Service Data Flows (SDFs). The EPS bearer is the minimum level of granularity at which QoS, mobility and security are provided within EPS, i.e., SDFs mapped to the same EPS bearer are treated with the same QoS, mobility and security policy. When a UE attaches to a PDN, after authentication, it is allocated an IP address and an EPS bearer is established. The EPS bearer remains established throughout the lifetime of the PDN connection to provide the UE with Always-ON IP connectivity. The bearer established at the attachment is referred to as the default bearer that provides default QoS, determined by the network upon UE subscriber data. Any additional EPS bearer that is established for the attached UE to the same PDN is referred to as a dedicated bearer, established when the UE issues a Service Request or when the network triggers a Service Request, for which a dedicated QoS treatment needs to be provided.

**Figure 1:**
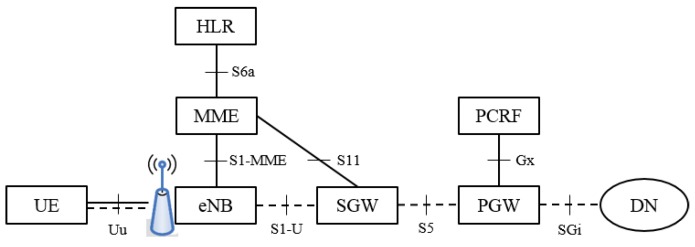
4G System Reference Architecture. HLR: Home Location Register; MME: Mobility Management Entity; eNB: evolved NodeB; SGW: Serving GateWay; PGW: Packet GateWay; PCRF: policy and charging rules function; DN: Data Networks; UE: User Equipment. The terminologies used here are all the 4G standard terminologies.

The EPS bearers are established by LTE/EPC CP, and they are E2E concepts: they provide connectivity from UE to the PLMN external PDN, hence traversing UP elements of RAN and CN. In particular, the EPS bearer is composed by the concatenation of the Radio bearer (between the UE and the access node), the S1 bearer (between the access node and the Serving GateWay, SGW) and S5/S8 bearer (between SGW and Packet Data Gateway, PGW). The 4G system architecture is depicted in Figure 1.

### 3.2. RAN Consideration

The RAN segment offers radio resources to devices to transmit their data. When coming to mIoT, the RAN has an important role from a CP point of view as devices perform the random access (RA) procedure to establish the bearer to carry data. While mIoT UP traffic requires a small amount of radio resources due to the transmission of small and infrequent packets, the RA might represent the bottleneck and for this reason it has been object of several enhancements to improve its capacity and efficiency. Referring to the current RA as in LTE, usually the amount of resources (i.e., a pre-defined set of pseudo-orthogonal preambles) for the RA is composed of 54 preambles available every 5 ms, which brings to a capacity of 10,800 RA resources per second. Obviously, collisions limit this capacity but it is worth noticing that collisions happen only when two or more devices select the same preamble in the same RA opportunity and this is obtained only for very high load scenario. For instance, by considering a RA periodicity of 5 ms, a heavy load of 10^3^ devices transmitting in 1 s (as for instance generated through a beta distribution as discussed in Section 2.2) brings to five devices per RA opportunity in LTE with a collision probability lower than 10%. In the case of huger load, congestion can be handled by means of the access class barring (ACB) [20] where a backoff mechanism is exploited before the preamble transmission to avoid a high number of devices performing the RA in the same RA opportunity. To further increase the capacity of the RA, Thomsen et al. [21] proposed the code-expanded approach where the RA is performed by transmitting a code-word composed of two or more preambles instead of a single one, thus drastically reducing the probability for two devices to select the same code-word. A more detailed survey on the works on RAN can be found in [22,23] with additional solutions to reduce the congestion in the RAN. Such works testify the interest of research community in increasing the capacity on the RAN and showing the feasibility of different solutions over LTE to handle mIoT traffic. An equivalent effort as surveyed in [22,23] and references therein does not have its counterpart in terms of analysis on the CP signaling, and this further motivates the novelty of the work carried out in this paper. 

A further aspect testifying the effectiveness of the enhancements on the access of 4G systems is that the RA in 5G systems (as well as in IoT-tailored technologies such as NB-IoT (Narrow Band IoT) and LTE-M (LTE for machine-type communications) is strongly based on the solutions developed for LTE. This underlines that the access is not considered as a segment requiring major enhancements towards the support of mIoT use cases in 5G. Indeed, the main issues in effectively handling the mIoT slice comes from the design of E2E solutions that need to consider the reduction of the CP signaling to support scalability. From this point of view, this work fills this gap proposing an effective solution to limit the CP signaling in the CN.

It is worth underling another aspect related to the role of the RAN (and in particular of the RA procedure) in controlling the CP/UP load towards the CN in case of overload. Radio overload means that there are not enough RA resources to handle all devices trying to connect to the network. Thus, a large portion of these devices will not be able to perform the RA procedure due to collisions. Consequently, CP/UP traffic from the RAN towards the CN will be reduced (this aspect will be analyzed in Sec. V.B of this paper). This means that, currently, the CN design takes advantage of this aspect as the CP signaling is expected to be low bounded by the RAN capacity. Nevertheless, increasing the capacity is a main target of 5G radio, which thus dictates for solutions to proper handle CP signaling to avoid overload in the CP. For this reason, we focus our attention on how to effectively reduce the CP signaling for mIoT.

### 3.3. EPC Consideration

An assessment of the EPS connectivity model applied to the mIoT use case highlights severe drawbacks in the CN. If, on the one hand, the sometimes strict mIoT QoS requirements may favor a EPS connection-oriented model, on the other hand, the sporadic and limited data transmission of mIoT devices and the consequent CP vs UP traffic ratio makes the Always-ON concept questionable.

First, default bearer establishment requires time, computation and storage resources for CP (eNB (evolved NodeB) and MME (Mobility Management Entity)) and UP (eNB-SGW, S1, and SGW-PGW, S5) entities. EPC network elements can simultaneously handle only a target maximum number of bearers (~10^6^–10^7^) and their complexity and cost increase accordingly. Supporting the massive CP load and handling bearers on UP even for initial mIoT scenarios on carrier grade would require either enhanced (and more expensive) EPC network elements, or network re-engineering, re-planning and re-deployment. Even with an approximate quantitative evaluation, we may estimate a thousand LTE cells to lead to up to one billion mIoT devices EPS attached (idle most of the time) and assuming best practice 4G network topologies, this goes clearly beyond EPC network element capability. 

In addition, even assuming enhanced CP and UP capable of handling the required number of bearers at core network side, the approach would lead to an extremely inefficient resource utilization at EPC. The Always-ON concept presumes the UE, while attached, will require frequent data exchange. This justifies the resources allocated to support the default bearer. Such assumption clearly does not apply to mIoT, where small and infrequent data transmission appears to be the dominant model. Consequently, a novel solution is needed to reduce the signaling. A possible approach has been proposed in [13], which introduces the concept of connection-less transmissions where devices transmit packets without any bearer establishment. The paper claims the gain relating to a connectionless access can be estimated evaluating the overhead reduction. In a first approximation, according to [13], connectionless access may lead to an overhead reduction of up to an order of magnitude, on both UL and downlink (DL). Although having a clear benefit from an access signaling reduction point of view, the lack of bearers involves the impossibility of handling QoS (thus limiting the applicability from a use case perspective). Moreover, it should be stressed that, again, the solution included in [13] focuses on RAN only, and does not consider at all the issue relating to massive connectivity, yet QoS constrained, at Core Network.

This short 4G EPC gap analysis suggests guidelines for the definition of the Core Network part of the 5G mIoT E2E Slice architecture and procedures: the need to provision QoS favors the adoption of a connection oriented connectivity model, while the expected traffic patterns clearly highlight the “Always-ON” principle should be superseded to allow efficient CP signaling and UP resources utilization. 

The next Section will focus on the design of a 5G mIoT slice able to support above mentioned goals through an enhanced bearer-oriented approach.

## 4. Introducing mIoT in 5G Systems

### 4.1. The 5G System Architecture

**Figure 2:**
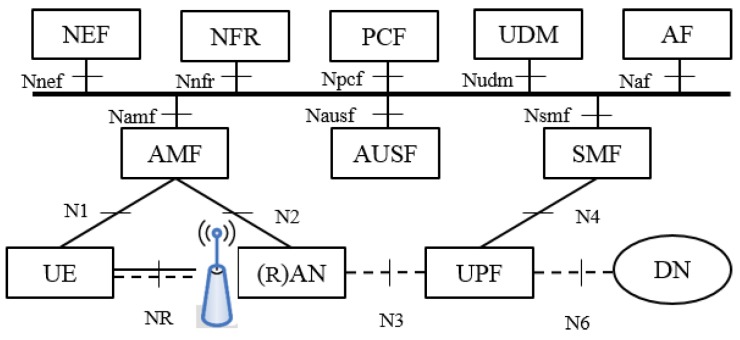
5G System Reference Architecture. NEF: Network Exposure Function; NRF: Network function Repository Function; PCF: Policy Control Function; UDM: Unified Data Management; AF: Application Function; AMF: core Access and Mobility management Function; AUSF: Authentication Server Function; SMF: Session Management Function; UE: User Equipment; (R)AN: (Radio) Access Network; UPF: User Plane Function; DN: Data Network.

The definition of a customized CP and UP to efficiently support mIoT services, based on an innovative connectivity model overcoming 4G limitations, is made possible within 5G system (5GS) thank to the architecture modularization and the support of network slicing. Figure 2 shows the 5GS reference architecture (as agreed by 3GPP in [14]). 5GS has been decomposed into a set of Network Functions (NFs), which can communicate either via point-to-point interfaces or via service-oriented interfaces, the latter illustrated in the figure. Without entering into excessive details, it is essential to highlight the role of some NFs. The Access and Mobility function (AMF) terminates the CP interfaces with the (R)AN (N2) and with the UE (N1), and manages UE registration, mobility, reachability and security. The Session Management function (SMF) manages the UE connectivity towards the User Plane functions (UPFs), which terminate the UP interface towards the (R)AN (N3), act as mobility anchor, and terminate the interface towards external Data Networks (N6).

Unlike 4G system, for which a single and monolithic architecture is defined, 5GS can support different network slices, i.e., different complete logical networks whose different architectures are defined by the interconnection of different set of customized CP and UP NFs. Network slices are defined “end to end” (i.e., including access and core network) and hence the customization of NFs can relate also to the access network: network slices can be differentiated at the access and the “R” between brackets aims to stress different access networks can be integrated in 5GS, including legacy RANs and non-cellular networks (e.g., non-3GPP access). Different network slices can be deployed over different infrastructure and can be independently accessed by different devices. When registering to the 5G network, UEs shall indicate the network slice(s) they will request service(s) from, and they will be authorized or denied the registration according to their credentials. Upon a service request for a given network slice from a UE, the AMF will route CP signaling to the CP NFs of the concerned slice and, likewise, a UP connection for the concerned network slice will be established including the relating (R)AN and UPF(s). This way, CP and UP will be fully customized on per network slice basis, via a dedicate design of the relating CP NFs and UPFs.

Within this reference framework, crystalized in 3GPP release 15 specification [14], multiple challenges will stem out in the coming years, i.e., how 5G architecture and procedures shall look for each network slice, to optimize performance, network efficiency and resource utilization for the related use cases.

Within the 5GS reference architecture, an mIoT network slice has been designed and is described in the following sections, overcoming the key limitations of 4G systems. It is important to observe that for 5GS defined by 3GPP release 15, commonly denoted as 5G phase 1, only eMBB slices are specified. mIoT slices will be specified by 3GPP release 16, for which the solution proposed by this paper could be a possible candidate. The design is based on the concepts of Virtual Device and Aggregate Core Network Bearer, which are implemented via a proper customization of AMF and SMF, as described in Section 4.2.

### 4.2. 5GS mIoT Slice Design

The design of the mIoT Network Slice for 5GS starts from the need for an innovative connection-oriented model allowing, on the one hand, to satisfy QoS requirements of mIoT services and, one the other hand, to efficiently serve massive number of connections both from CP and UP perspective. Consistently with the gap analysis described in Section 3, major enhancements are proposed on the core network side while, to handle deployment scenarios and traffic models as per Section 2, a proper configuration of 4G LTE is considered sufficient as access network.

To achieve the design goals, some new concepts are introduced. First, the device class for PhDs, which identifies the communication requirements of clusters of physical devices (e.g., QoS profile, min Reliability, min Availability, supported PDN, and PDN specific requirements). PhDs belonging to the same device class are characterized by homogeneous communication requirements and hence, their data transmission requires a homogeneous treatment by the network. Second, the Virtual Device (VD) concept is introduced: a VD is a logical entity at CN side which corresponds to the aggregation of some (or all) PhDs camped on the same cell and belonging to the same device class, i.e., a multitude of PhDs belonging to the same device class are handled at CN as a single VD, whose behavior are regulated by a single state machine (like EMM and ECM state machine regulate 4G UEs). For a given device class, a maximum number U of PhDs can be associated to a VD. A VD inherits the same device class of the PhDs it is it is meant to gather.

Finally, the concept of Aggregate Core Network (ACN) bearer is introduced, transporting UP data from the (R)AN UP function through the Core Network UPFs until the external PDN. An ACN bearer is associated to each VD, and it transports data from the (R)AN/PDN Gateway to the PDN Gateway/(R)AN generated by/from all PhDs composing the VD. The ACN bearer inherits the device class of the corresponding VD. In other words, the ACN bearer makes a single simplified UP for a plurality of PhDs for which data packets require the same treatment. For a given Device Class, each ACN bearer is associated to an activity time T, restarted at each data transmission. Upon T expiration, the ACN bearer is released and the VD is de-allocated.

The defined Device Class, Virtual Device and Aggregate Core Network Bearer concepts are the cornerstones for the definition of a 5G mIoT network slice. From a pure architecture perspective, the proposed mIoT slice shall be composed by the interconnection of all the basic NFs defined for 5GS. Compared to 4G LTE/EPC system and to the 5G eMBB slice defined in [11], the proposed mIoT slice differs in the CP procedures for device registration and session set up, as well as for the user plane model. Briefly, all PhDs of a given Device Class camped on the same access node are associated to a single VD, for which a single ACB from (R)AN to UPF exists to transfer UP data. Saved for the first PhD registering to the mIoT network slice, at each PhD registration, there is no need to establish any UP connection, as the registering PhD is simply associated to the VD of the corresponding Device Class, and its UP data can be carried (both UL and DL) over the ACB previously established for the VD. Hence, through a modified registration and bearer set up procedure, the concept of Device Class, Virtual Device and Aggregate Core Network Bearer can be implemented in the mIoT slice, this affecting primarily the AMF and SMF functions. It should be noted that, on access side, mIoT slice procedures at device registration are assumed the same as per the eMBB slice, as defined in [24]. As discussed in detail in Section 5.2, compared to 4G, the mIoT slice solution reduces the amount and complexity of CP signaling, as it allows to avoid the establishment of a default bearer per each PhD. Moreover, by multiplexing data of multiple PhDs into the same ACB, the solution clearly aims at a more efficient UP resource utilization.

It worth noting the proposed solution, once standardized, would allow a seamless deployment of mIoT slices within any early real 5G system. Assuming a reasonable reference scenario where a Release 15 compliant 5G System has been set up (to provide eMBB service) via the deployment of NR access nodes connected to a 5GC instantiated over general purpose cloud infrastructure, the deployment of an mIoT slice would substantially require three basic steps: first, a software upgrade of the access nodes which would be supposed to allow access of the mIoT devices, to support the aggregate core network bearer towards UPFs; second, a software update of the AMF, to enable the handling of the simplified mIoT CP procedures; and, finally, the deployment of mIoT specific SMF instances, dedicated to the handling of the UP for the related slice. Obviously, other 5GC network functions would require configuration updates (e.g., the NRF and NEF, to allow the services provided by mIoT specific SMF to be accessible by other 5GC NFs); these operational details, however, are out of the scope of this work, as not directly relating to architecture design. Similar steps would of course apply for the deployment of newly defined network slices.

Standard compliant 5G mIoT devices, while accessing the network for registration, will provide the identifier of the mIoT slice they require service from (i.e., S-NSSAI (Single Network Slice Selection Assistance Information), as specified in [14]), this enabling the AMF to route CP signaling to the suitable SMF providing the coherent CP and UP handling.

**Figure 3:**
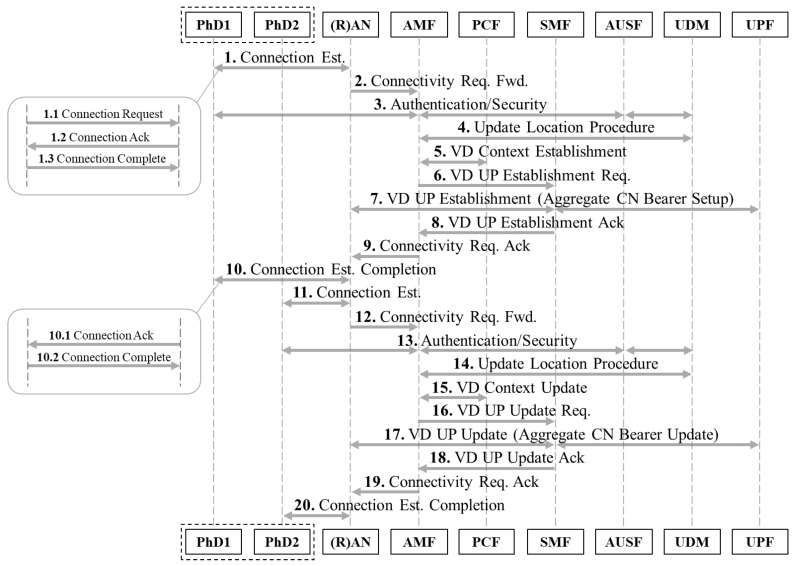
Physical Device Registration and Aggregate Core Network (ACN) bearer Setup/Update. VD: Virtual Device; UP: User planes; CN: core network.

Operational details of the 5GS mIoT network slice are illustrated in Figure 3. The description of the procedure on the access side is simplified for brevity, and as no major differences exist compared to 4G systems. Detailed description of Dteps 1 and 10 is included in [20] for 4G systems and in [24] for 5GS. At the registration of the first PhD of a given device class, after successful authentication (Step 3), the context for a class C Virtual Device is created at AMF (Step 5) and a virtual address is allocated to the VD. Subsequently, the SMF establishes an ACN bearer between the UPF of the (R)AN the PhD accessed and all the UPFs required to route data to the external PDN (Step 7). At the registration of (up to U, where U can vary for each device class) further PhDs of the same class and accessing from the same (R)AN node, after successful authentication (Step 13), such devices are associated to the same VD (Steps 15–17) and their data will be transported from/to the PDN via the correspondent ACN bearer previously established. The SMF executes an update of the bearer, to ensure proper data routing for all PhDs. It should be noted a bearer update will require less CP resource compared to a bearer setup.

Although different PhD addressing schemes will be envisioned (and they are out of scope of this work), it is assumed each mIoT data packet is associated to a source address and a destination address, and it is encapsulated into the SDU (service data unit) transported by the ACN bearer. For UL transmission, the virtual address of the VD is used to route packets generated from all PhDs to the PDN UPF Gateway; packets will be further forwarded within the external PDN based on the destination address of the packet generated by the PhDs. For DL transmission, incoming packets at PDN UPF Gateway associated to a VD can be routed to the appropriate (R)AN UPF node; the (R)AN node, however, requires additional information to identify the PhD the packet is directed to. To this end, the aggregate bearer SDUs will have to be tagged at PDN UPF Gateway according to the packet destination address; based on the tag, the (R)AN node will be enabled to address the proper physical device on the radio link. The ACN bearer update is needed to enable the tagging at PDN UPF Gateway and the tag identification at (R)AN.

## 5. Performance Evaluations

### 5.1. Solution KPIs and Simulation Scenarios

We perform a quantitative evaluation of our proposed 5G mIoT network slice, and compare its performance with a baseline 4G solution. The analysis has been focusing on the CP signaling and UP, taking the followings into consideration:

- *total CP signaling*, measured in CP msg/s. It is the overall CP signaling in the CN given by the RAN-CN signaling (i.e., between eNodeB and MME in 4G networks and between the RAN and the AMF when considering 5GS) plus the signaling within the CN (i.e., between the MME and the SGW/PGW in 4G networks and among the AMF, SMF and UPF in 5GS).
- *bearer establishment*, measured in CP msg/s. It is the component of the CP signaling related to EPS/ACN bearer establishment that is triggered by the MME in 4G networks and by AMF for 5GS.
- *bearer re-setup* measured in CP msg/s (We are looking into a different method of bearer establishment and re-setup, as these messages could involve a different load for the CP entities.). It is the component of the CP signaling related to EPS/ACN re-setup, i.e., when an already existing bearer has been de-activated due to UP inactivity and needs to be activated again.
- *bearer update*, measured in CP msg/s. It is considered only for the 5GS mIoT slice and it is the component of the CP signaling related to the update of an ACN bearer when a new PhD is added to an existing ACN bearer (i.e., Step 16 in Figure 3).
- *UP resource utilization*, measured in UP pkt/bearer, i.e., the ratio between the UP traffic and the bearers enabled by the network to handle such traffic.

The evaluation was performed using a software tool developed in Matlab simulation environment. An analogous network deployment for 4G and 5G systems has been considered, assuming that access nodes are connected to a single CN instance (a single MME, SGW/PGW for 4G, and a single AMF, SMF, UPF, PDN Gateway for 5G). In the developed software, devices perform a random access (RA) procedure [22] before transmitting their data. The random access procedure has been implemented by considering aspects such as power ramping, preamble collision as well as capacity limitation for random access response (RAR). RAN nodes have been configured as in [17] (macro-cellular coverage with ISD (inter site distance) = 1.732 km, 2 GHz carrier frequency, 5 MHz bandwidth, 54 preambles, RA periodicity set to 5 ms, 10 maximum RA attempts, and maximum six preambles addressed in a single RAR message). More details on simulation assumptions can be found in [17,25]. The implemented simulation environment provides as outputs the CP signaling and the UP traffic generated from the random access procedure. The random access has been implemented by considering the signaling as discussed in [20] with the related parameters affecting the capacity of the cell and the latency in the generation of control and data messages (i.e., amount of time and frequency resources, parametrization of control and data channels, priority of data and control messages). In more details, the simulation environment models PRACH (Physical Random Access Channel), PDCCH (Physical Downlink Control Channel), PDSCH (Physical Downlink Shared Channel), and PUSCH (Physical Uplink Shared Channel) with the related RRC (Radio Resource Control) signaling generated between the UE and the base station and between the base station and the CN. The UP traffic is defined with packet size of 300 Bytes [26], while density and activation period (AP) of devices has been varied in our analysis. We considered a simulation interval of 1 h.

### 5.2. Simulation Results and Discussion

**Figure 4:**
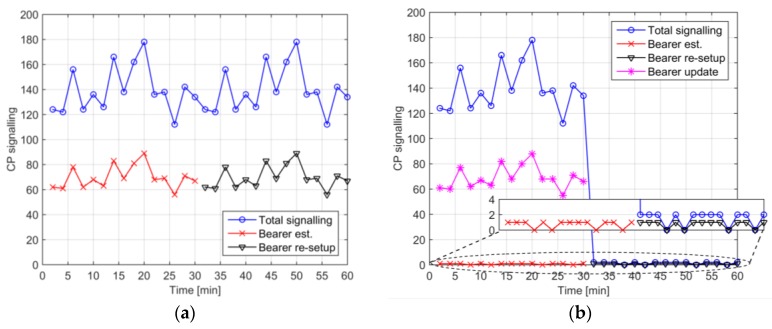
(**a**) Time variation of CP signaling in 4G; and (**b**) time variation of CP signaling with 5GS mIoT (U = 100 and T = 1 min). The analysis considered AP = 30 min, 1 RAN node and 10^3^ UE/km^2^.

In Figure 4, we focus our attention on the variation of the total CP signaling during the simulation interval of interest and on its components related to bearer establishment, re-setup and update. For the sake of simplicity, in this analysis, we consider a single access node. We also assume a density of 10^3^ UE/km^2^ and that all UEs have an AP = 30 min. Observing the time evolution of the CP signaling on the left-hand side in 4G, we can note that bearers are established during the first half interval of the simulation. In 4G, this requires one bearer establishment for each PhD. In the 5GS mIoT slice, bearer establishments are needed only for VDs which is a much lower number compared to PhDs (this depends on the ratio between the number of PhDs and U (Maximum number of PhDs for a VD)) while the remainder of CP signaling is related to bearer updates (which involved less load for the CP). Once bearers are established, the CP traffic is related to re-setup when PhDs transmit other traffic. In 4G, the load of re-setup is the same as in the establishment phase (i.e., one re-setup for each PhD) and this means that the load towards the CN in 4G does not significantly change during establishment and re-setup phases. Although re-setup might involve less load, the number of CP messages is the same as for the establishment, i.e., the re-setup in 4G involves the same CP msg/s as for the establishment phase. With 5GS mIoT slice, only one re-setup for each VD is needed, i.e., the re-setup of one aggregate core network bearer does not require bearer updates as the bearer has been already established. This means that after the establishment phase, the CP signaling is very limited in the 5GS mIoT slice. Another aspect to underline is that the load due to re-setup can be tuned with a different strategy when creating the VDs. In our analysis, VDs are created based on the traffic pattern, i.e., two consecutive transmitting PhDs are associated to the same VD if the VD has still enough space (i.e., U is not exceeded). This means that all PhDs gathered in a VD transmit their UP traffic close to each other, thus the aggregate core network bearer associated to the VD is detached due to UP inactivity and then re-setup. The PhD-VD association could be enhanced by considering application (e.g., traffic patterns) and UE (e.g., density) contexts to avoid the expiration of an aggregate core network bearer thus further reducing the CP signaling. Possible strategies could be to associate consecutive transmitting PhDs to different VDs or to add a new PhD to an existing VD only when the lifetime related to the VD is close to expire.

**Figure 5:**
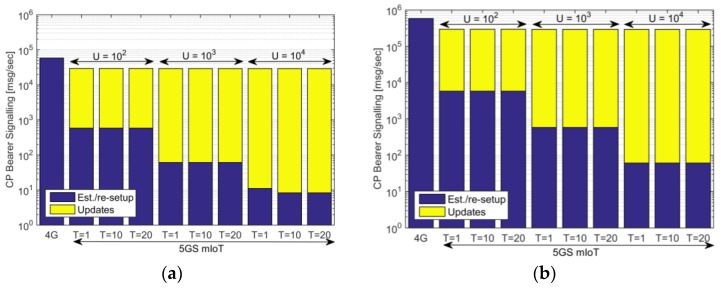
(**a**) CP bearer signaling with 10^4^ UE/km^2^; and (**b**) CP bearer signaling with 10^5^ UE/km^2^. The analysis considered AP = 30 min, 10^4^ RAN nodes and different values of U (Maximum number of PhDs for a VD) and T (Bearer Inactivity Timer) for the 5GS mIoT.

After the analysis of the time variation of the CP signaling generated by a single access node, the evaluation will now consider a closer to reality scenario with 10^4^ RAN nodes connected to the same CN. To understand the behavior of the two approaches discussed in this paper, in this analysis, the UE density is varied (the AP is 30 min) and, for the 5GS mIoT, U and T parameters have been varied to analyze their impact on the achieved performance. Parameters used for this analysis are summarized in Table 1. In Figure 5, we analyze the CP signaling related to bearer management, and study the compositions of the different components. In this analysis, establishments and re-setups are gathered together for the sake of simplicity, but given the fact that AP = 30 min and the interval of interest is 1 h, establishments and re-setup are equally split: UEs transmit on average two packets in the simulation period of 1 h, where the first transmission requires a bearer establishment, while the second transmission only asks for bearer re-setup. The 5GS mIoT introduces a reduction of ~50% for the CP bearer signaling compared to 4G for this traffic pattern. This is because the CP signaling for the 5GS mIoT is mainly due in the initial phase of the traffic (establishment and updates, as seen in Figure 4), the re-setup phase involves a low CP signaling as performed on a VD-basis, i.e., the number of re-setups depends on the number of VDs and not on the number of PhDs. It is interesting to note that the reduction related to bearer signaling does not significantly vary when increasing U or T (Bearer Inactivity Timer). In particular, T does not influence the CP signaling in the considered scenario, while U has a very low impact: the case with device density of 10^4^ UE/km^2^, U = 10^2^ and U = 10^4^ introduce a CP signaling reduction with respect to 4G of ~49% and ~53%, respectively. This is because, when increasing U, the number of establishment/re-setup/updates is slightly modified and, thus, the CP signaling reduction is almost unaffected. Another aspect to highlight in Figure 5 is the composition of the CP signaling for the 5GS mIoT, which highlights how the signaling is mainly composed of bearer updates and this increases with the value of U. Focusing on the scenario with a device density of 10^4^ UE/km^2^, the difference between establishment/re-setup and setup varies from one to four orders of magnitude for U = 10^2^ to four U = 10^4^, respectively. Considering that updates involve less load compared to establishment and re-setup, this analysis underlines how effective is the 5GS mIoT solution in reducing the CP load in the CN, as increasing the value of U brings the benefit of reducing the number of establishments and re-setup.

**Figure 6:**
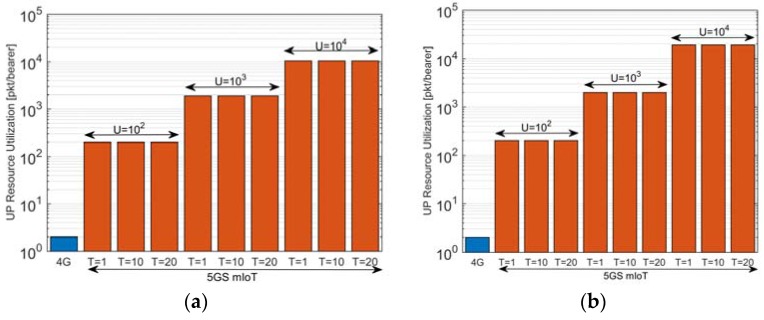
(**a**) UP Resource Utilization with 10^4^ UE/km^2^; and (**b**) UP Resource Utilization with 10^5^ UE/km^2^. The analysis considered AP = 30 min, 10^4^ RAN nodes and different values of U and T for the 5GS mIoT.

A further analysis of the impact of U can be seen in Figure 6, which plots the UP resource utilization for the scenario in Table 1. The higher is U, the higher is the number of PhDs re-using the same aggregate core network bearer and thus the higher is the resource utilization. It is thus clear that the size of U has a clear impact on the UP resource utilization. Although it may seem immediate that using higher U increases the benefits introduced by the 5GS mIoT, it is worth underlining that higher U means higher number of PhDs and this might mean higher UP resource request (i.e., bandwidth) ACN bearer in case the network is required to simultaneously support the transmission by multiple PhDs of the same VD. This means that tuning U may depend on the available resources for the UP (i.e., network context): using higher U if enough UP resources are available or lower U on the contrary. High U may also introduce addressing issues as high large tag size requirements resulting in increased packet overhead. Tuning of U, hence, should consider the above inverse performance impacts. To summarize the results in Figure 5 and Figure 6, a proper tuning of the inactivity timer T affects the ratio between the signaling related to ACN bearer establishment/re-setup and updates, with gains compared to 4G up to three orders of magnitude when increasing T considering the scenario in Table 1. A proper tuning of the maximum number of PhDs for a VD (U) positively impacts the UP resource utilization, with a gain with respect to 4G of three orders of magnitude when increasing U considering the scenario in Table 1.

After analyzing the behavior of 4G and 5GS mIoT in a specific scenario where all devices have the same AP, we now consider a realistic scenario with different classes of devices, each with different UE density and AP. We consider the traffic model in [26,27], which are summarized in Table 2. For 5GS mIoT, we assume that a VD gathers PhDs belonging to the same device class to reflect the fact that different traffic types may require different QoS treatment due to the nature of their traffic or due to the SLAs between the owners of the devices and the operator. For 5GS mIoT, all classes of devices have U = 100 and T = 1 min.

**Figure 7:**
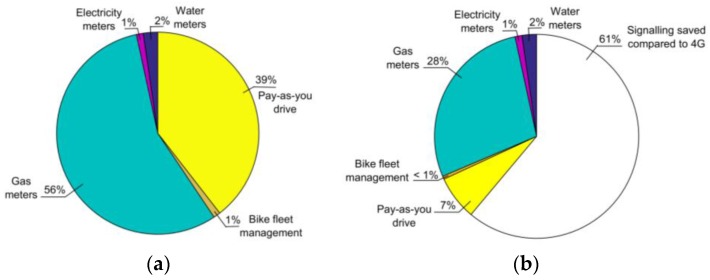
(**a**) CP signaling composition among different classes of devices in 4G; and (**b**) CP signaling composition in 5GS mIoT and reduction with respect to 4G. For 5GS mIoT, all classes of devices have U = 100 and T = 1 min.

**Figure 8:**
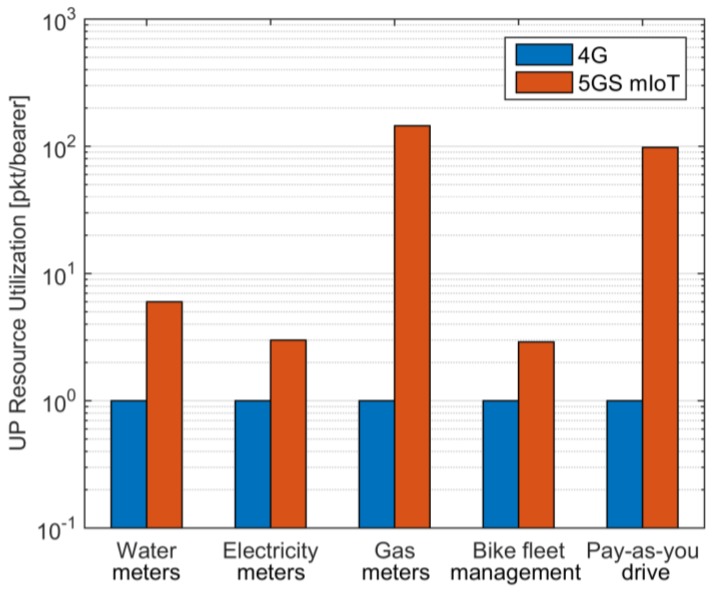
UP Resource Utilization among different classes of devices. For 5GS mIoT, all classes of devices have U = 100 and T = 1 min.

The analysis shows that 5GS mIoT slice is able to guarantee a CP signaling reduction of ~60% with respect to EPS (Figure 7) when considering a practical scenario with different types of active devices with an increase in the UP resource utilization up to 2 orders of magnitude (Figure 8). In addition, it is worth underlining the benefits introduced by the 5GS mIoT in reducing the impact with respect to 4G of applications with massive CP signaling (i.e., gas meters and pay-as-you-drive in the considered scenario) in both CP and UP. A further analysis can be found in Table 3, which lists the number of enabled bearers assuming a deployment of 10^4^ RAN nodes. The 5GS mIoT slice has on average a reduction of about two orders of magnitude in the number of enabled bearers compared to 4G, which further testifies the reduction of CP load in the CN introduced by the 5GS mIoT slice. Given the fact the network entities in the CN can support only a limited number of bearers simultaneously active, 5GS mIoT is an effective solution to increase the number of supported UEs without requiring more capable (and thus expensive) network entities.

**Figure 9:**
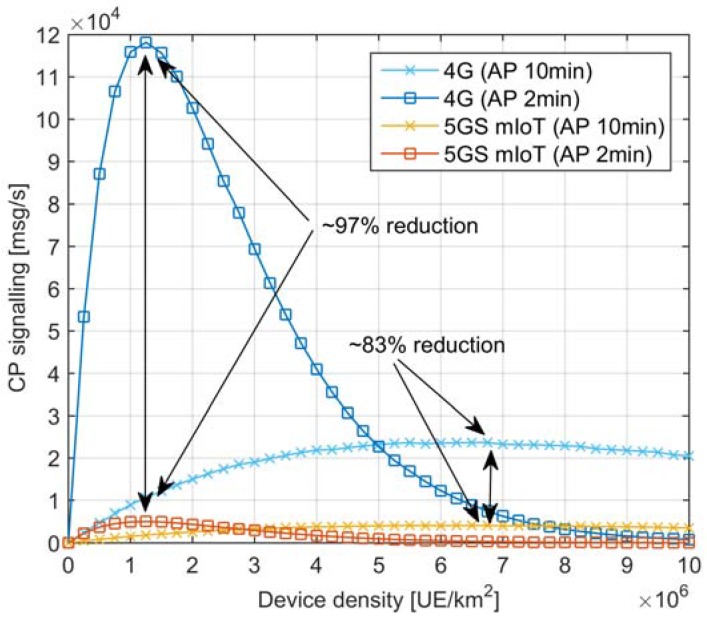
CP signaling varying the UE density to load the RAN (1 RAN node, all UEs belonging to the same class, AP = 2 min, U = 100 and T = 1 min).

Finally, the analysis in Figure 9 aims at understanding the CP signaling generated with 4G systems and the related benefits introduced by the 5GS mIoT in scenarios with massive load on the RAN, as envisaged for 5G mIoT use cases with up to millions of IoT devices per cell. To this end, an analysis has been conducted varying the device density up to 10 × 10^6^ UE/km^2^. To better isolate the behavior of the RAN, only one RAN node is considered. All UEs are assumed to belong to the same class, where AP = 2 min and AP = 10 min were considered. The bearer expiration timer is T = 1 min. Finally, for 5GS mIoT, the maximum number of PhDs per VD is U = 100. From this analysis, we can note that the CP signaling reaches a maximum value before starting the congestion, i.e., high collision in the RA procedure. When the device density increases, the CP signaling goes close to zero as a very large portion of devices is affected by RA collisions and it is not thus able to send a connection request towards the CN. It is worth noting that, when reaching the maximum load the radio can support (~7 × 10^6^ UE/km^2^ and ~10^6^ UE/km^2^ for AP = 10 min and AP = 2 min, respectively), 5GS mIoT slice reduces the signaling towards the CN of about 97% and 83% for AP = 10 min and AP = 2 min, respectively. This is because most of CP traffic is bearer re-setup which is a heavy load for 4GS while 5GS mIoT slice drastically reduces this signaling. Consequently, 5GS mIoT slice can also avoid CP overload in scenarios where the radio is overloaded. It is worth underlining that the analysis in Figure 9 considers only one RAN node, and that practical scenarios with thousands of RAN nodes connected to the same CN would generate an overall CP signaling from three to four orders of magnitude higher, thus collapsing the CN. The analysis represented in Figure 9 shows that 5GS mIoT is successfully designed to drastically reduce the CP signaling even in scenarios with massive load on the RAN, meaning that 5GS mIoT can effectively be adopted in 5G deployments with higher RAN capacity compared to 4G systems (thus generating more traffic towards the CN) without overloading the CN.

This means that 5GS mIoT slice could allow RAN nodes to work close to their maximum congestion levels without affecting the CP entities in the CN.

## 6. Conclusions

In this paper, the design and performance evaluation of an E2E mIoT network slice for 5G system has been presented. Starting from an analysis of the envisaged device deployment scenarios and the expected traffic models, the solution targeted requirements in terms of massive device deployments, sporadic connectivity, and small QoS-constrained data transmission. Assuming a 4G-like network deployment, the analysis highlighted the CN as the main system bottleneck for providing massive connectivity. Hence, the proposed E2E solution combined a mIoT enhanced LTE access with a 5GS mIoT featuring a new connectivity model. The new connectivity model is based on the virtual device and Aggregate Bearer concepts, together allowing the 5G core to handle multiple physical devices accessing the network via the same access node as a single logical element. The solution evaluation, still preliminary but considering relevant deployment scenarios and traffic models, has shown a 5GS CP signaling reduction up to 60% compared to a baseline LTE/EPC system when considering close-to-reality mIoT traffic, and the capability of dramatically improving the efficiency in UP resource utilization. Future work will focus on extending the performance evaluation to different mIoT device classes and diverse device deployment scenarios. Further device class specific optimizations will also be investigated.

## Figures and Tables

**Table 1:** Parameters for the analysis in Figure 5 and Figure 6.

Parameter	Value
RAN nodes	10^4^
UE activation period (AP)	30 min
UE density	10^4^ UE/km^2^, 10^5^ UE/km^2^
Maximum number of PhDs for a VD (U)	10^2^, 10^3^, 10^4^
Bearer Inactivity Timer (T)	1 min, 10 min, 20 min

**Table 2:** Traffic parameters of device class considered in the analysis in Figure 7 and Figure 8, and Table 3.

Device Class	Device Density	Activation Period (AP)
Water meters	10^4^ UE/km^2^	12 h
Electricity meters	10^4^ UE/km^2^	24 h
Gas meters	10^4^ UE/km^2^	30 min
Bike fleet management	200 UE/km^2^	30 min
Pay-as-you-drive	2250 UE/km^2^	10 min

**Table 3:** Number of enabled bearers for different classes of devices.

Traffic Type	Number of EPS Bearers (4G)	Number of ACN Bearers (5GS mIoT)
Water meters	~8.7 × 10^6^	~9 × 10^4^
Electricity meters	~4.3 × 10^6^	~5 × 10^4^
Gas meters	~1 × 10^8^	~1 × 10^6^
Bike fleet management	~2 × 10^6^	~3 × 10^4^
Pay-as-you-drive	~2.3 × 10^7^	~2.4 × 10^5^

EPS: Evolved Packet System; ACN: Aggregate Core Network.

## References

[1] (2016). 3GPP TR 22.891 V14.2.0 (2016-09), Technical Report; Feasibility Study on New Services and Markets Technology Enablers. https://portal.3gpp.org/desktopmodules/Specifications/SpecificationDetails.aspx?specificationId=2897.

[2] Mahmood K., Mahmoodi T., Trivisonno R., Gavras A., Trossen D., Liebsch M. On the integration of verticals through 5G control plane. Proceedings of the 2017 European Conference on Networks and Communications (EuCNC).

[3] Jiang M., Condoluci M., Mahmoodi T. Network slicing in 5G: An auction-based model. Proceedings of the 2017 IEEE International Conference on Communications (ICC).

[4] Zhang N., Yang P., Zhang S., Chen D., Zhuang W., Liang B., Shen X.S. (2017). Software Defined Networking Enabled Wireless Network Virtualization: Challenges and Solutions. IEEE Netw..

[5] Palattella M.R., Dohler M., Grieco A., Rizzo G., Torsner J., Engel T., Ladid L. (2016). Internet of Things in the 5G Era: Enablers, Architecture, and Business Models. IEEE J. Sel. Areas Commun..

[6] Al-Fuqaha A., Guizani M., Mohammadi M., Aledhari M., Ayyash M. (2015). Internet of Things: A Survey on Enabling Technologies, Protocols, and Applications. IEEE Commun. Surv. Tutor..

[7] Bockelmann C., Pratas N., Nikopour H., Au K., Svensson T., Stefanovic C., Popovski P., Dekorsy A. (2016). Massive machine-type communications in 5g: Physical and MAC-layer solutions. IEEE Commun. Mag..

[8] Choi J. (2016). On the Adaptive Determination of the Number of Preambles in RACH for MTC. IEEE Commun. Lett..

[9] Arouk O., Ksentini A., Taleb T. (2016). Group Paging-Based Energy Saving for Massive MTC Accesses in LTE and Beyond Networks. IEEE J. Sel. Areas Commun..

[10] Elsaadany M., Ali A., Hamouda W. (2017). Cellular LTE-A Technologies for the Future Internet-of-Things: Physical Layer Features and Challenges. IEEE Commun. Surv. Tutor..

[11] Jiang Z., Krishnamachari B., Zheng X., Zhou S., Ni Z. (2018). Timely Status Update in Massive IoT Systems: Decentralized Scheduling for Wireless Uplinks. arXiv.

[12] Biral A., Centenaro M., Zanella A., Vangelista L., Zorzi M. (2015). The challenges of M2M massive access in wireless cellular networks. Digit. Commun. Netw..

[13] Kahn C., Viswanathan H. (2015). Connectionless access for mobile cellular networks. IEEE Commun. Mag..

[14] 3GPP TS 23.501, v15.0.0, Technical Specification Group Services and System Aspects; System Architecture for the 5G System; Stage 2, (Release 15). https://portal.3gpp.org/desktopmodules/Specifications/SpecificationDetails.aspx?specificationId=3144.

[15] Bader A., ElSawy H., Gharbieh M., Alouini M.S., Adinoyi A., Alshaalan F. (2017). First Mile Challenges for Large-Scale IoT. IEEE Commun. Mag..

[16] (2016). 3GPP TR 22.861 V14.1.0 (2016-09), Technical Report, Technical Specification Group Services and System Aspects; Feasibility Study on New Services and Markets Technology Enablers for Massive Internet of Things. http://www.tech-invite.com/3m22/tinv-3gpp-22-861.html.

[17] 3GPP TR 37.868 V11.0.0 (2011-09); Technical Report; Technical Specification Group Radio Access Network; Study on RAN Improvements for Machine-Type Communications; (Release 11). http://www.qtc.jp/3GPP/Specs/37868-b00.pdf.

[18] 3GPP TR 36.888 V12.0.0 (2013-06); Technical Specification Group Radio Access Network; Study on Provision of Low-Cost Machine-Type Communications (MTC) User Equipments (UEs) Based on LTE (Release 12). http://www.tech-invite.com/3m36/tinv-3gpp-36-888.html.

[19] 3GPP TR 45.820 V13.1.0 (2015-11); Technical Report; Technical Specification Group GSM/EDGE Radio Access Network; Cellular System Support for Ultra-Low Complexity and Low Throughput Internet of Things (CIoT) (Release 13). https://portal.3gpp.org/desktopmodules/Specifications/SpecificationDetails.aspx?specificationId=2719.

[20] 3GPP TS 36.331, Evolved Universal Terrestrial Radio Access (E-UTRA); Radio Resource Control (RRC). https://portal.3gpp.org/desktopmodules/Specifications/SpecificationDetails.aspx?specificationId=2440.

[21] Thomsen H., Pratas N.K., Stefanovic C., Popovski P. (2013). Code-expanded radio access protocol for machine-to-machine communications. Trans. Emerg. Telecommun. Technol..

[22] Laya A., Alonso L., Alonso-Zarate J. (2013). Is the Random Access Channel of LTE and LTE-A Suitable for M2M Communications? A Survey of Alternatives. IEEE Commun. Surv. Tutor..

[23] Ghavimi F., Chen H.H. (2015). M2M Communications in 3GPP LTE/LTE-A Networks: Architectures, Service Requirements, Challenges, and Applications. IEEE Commun. Surv. Tutor..

[24] 3GPP TS 38.331 V15.0.0 (2017-12); NR Radio Resource Control (RRC) Protocol Specification, (Release 15). http://www.tech-invite.com/3m38/tinv-3gpp-38-331.html.

[25] Condoluci M., Araniti G., Mahmoodi T., Dohler M. (2016). Enabling the IoT Machine *Age with 5G: Machine-Type* Multicast Services for Innovative Real-Time Applications. IEEE Access.

[26] Orange, Ericsson Traffic Model for Legacy GPRS MTC” (February 15–19, 2016), *Document GP 160060*, 3*G*PP GERAN meeting #69. https://www.ericsson.com/assets/local/mobility-report/documents/2016/ericsson-mobility-report-november-2016.pdf.

[27] (2016). Ericsson, Massive IoT in the City. https://www.ericsson.com/assets/local/mobility-report/documents/2016/emr-november-2016-massive-iot-in-the-city.pdf.

